# Postoperative atrial fibrillation is associated with long-term morbidity and mortality in older adults: Analysis from the SWEDEHEART Registry

**DOI:** 10.1016/j.xjon.2024.03.001

**Published:** 2024-03-12

**Authors:** Mathias Lilja, Richard Leaback, Jonas Banefelt, Tae Jin Park, Darshini Shah, William G. Ferguson, Örjan Friberg

**Affiliations:** a Quantify Research AB, Stockholm, Sweden; b AbbVie, Marlow, United Kingdom; c AbbVie, Irvine, Calif; d AbbVie, Madison, NJ; e Department of Cardiothoracic and Vascular Surgery, Faculty of Medicine and Health, Örebro University, Örebro, Sweden

**Keywords:** cardiac surgery, postoperative atrial fibrillation, morbidity, mortality

## Abstract

**Objectives:**

Postoperative atrial fibrillation (POAF) is the most common perioperative arrhythmia. The association of POAF with negative short-term outcomes after cardiac surgery is well understood; however, the association of POAF with long-term morbidity and mortality is not well described. We compared the risk of long-term clinical outcomes (up to 9 years postdischarge) in patients with and without POAF following open-chest cardiac surgery.

**Methods:**

This observational, retrospective cohort study used data from the Swedish Web-system for Enhancement and Development of Evidence-based care in Heart disease Evaluated According to Recommended Therapies (SWEDEHEART) Swedish Cardiac Surgery Registry and National Board of Health and Welfare. Patients aged 55 to 90 years who underwent open-chest coronary artery bypass and/or valvular surgery between 2010 and 2019 were included. Clinical outcomes were adjusted for differences in baseline demographics and clinical history using multivariable Cox regression.

**Results:**

A total of 30,870 patients with a mean age of 69.2 years were included in the study (no POAF, n = 20,734; POAF, n = 10,136). The median follow-up was 4.6 years. After adjustment, POAF was associated with a significantly higher risk of recurrent atrial fibrillation (hazard ratio [HR], 2.30; 95% CI, 2.21-2.41), heart failure (HR, 1.17; 95% CI, 1.10-1.25), chronic kidney disease (HR, 1.15; 95% CI, 1.07-1.24), all-cause mortality (HR, 1.11; 95% CI, 1.04-1.18), and cardiovascular mortality (HR, 1.16; 95% CI, 1.06-1.26). POAF was also associated with a numerically higher risk of ischemic stroke and major bleed, but these findings were not statistically significant after adjustment.

**Conclusions:**

These data provide further insight into the long-term clinical outcomes associated with POAF in patients undergoing cardiac surgery.

## Graphical abstract

**Figure:**
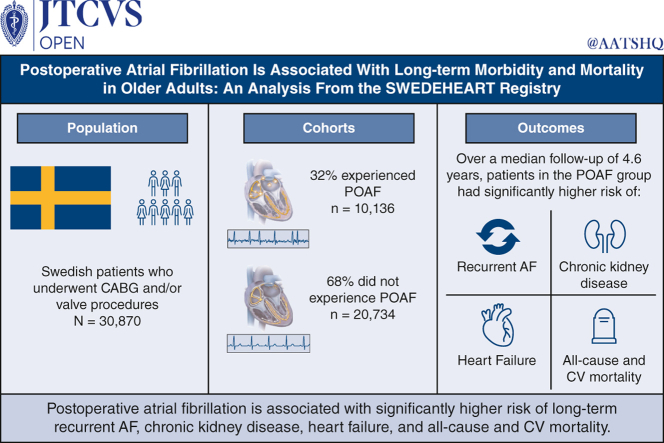

**Figure:**
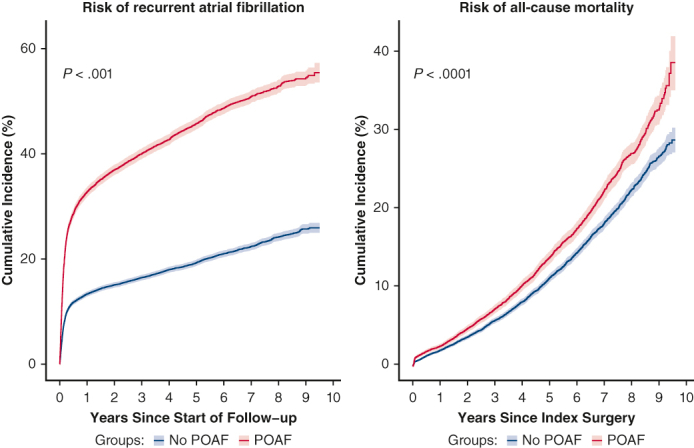
POAF is associated with long-term morbidity and mortality in older adults.

**Central Message**

Postoperative atrial fibrillation is associated with increased risk of long-term negative clinical outcomes for patients undergoing open-chest cardiac surgery.

**Perspective**

Postoperative atrial fibrillation (POAF) is the most common perioperative arrhythmia; however, the association of POAF with long-term morbidity and mortality is not well described. Leveraging comprehensive real-world data from the Swedish Cardiac Surgery Registry, this study reports increased risk of negative clinical outcomes associated with POAF over a median follow-up period of 4.6 years.

Postoperative atrial fibrillation (POAF) is the most common perioperative arrhythmia.^1^ The condition is characterized by intermittent episodes of atrial fibrillation (AF) typically occurring within 4 days of surgery, with incidence peaking at day 2.^2^^,^^3^ POAF occurs in approximately 35% of patients undergoing cardiac surgery, with rates increasing with surgical invasiveness and highest rates reported after valvular surgery.^3^, ^4^, ^5^ Ultimately, 25% to 70% of patients who experience POAF progress to recurrent AF, which is associated with negative outcomes after cardiac surgery.^6^, ^7^, ^8^ POAF that occurs following cardiac surgery is associated with longer hospital stays and higher health care resource utilization.^3^^,^^9^, ^10^, ^11^, ^12^, ^13^ In addition, POAF can more than double the risk of stroke and can lead to serious postoperative complications, including cardiac arrest and increased risk of mortality within 30 days and 6 months of surgery.^3^^,^^14^

Despite decades of advancement in cardiac surgery, the incidence of POAF has not improved, and the prevalence of many POAF risk factors is increasing.^3^ Risk factors for POAF may include age, prior history of arrhythmias, congestive heart failure (HF), coronary artery disease, hypertension, smoking, high cholesterol, chronic kidney disease (CKD), diabetes, and obesity, among others.^3^^,^^4^ Much of the current literature focuses on the short-term consequences of POAF, whereas long-term clinical outcomes such as morbidity and mortality are not as well described.^8^

The Swedish Cardiac Surgery Registry was formed in 1992 and covers all cardiac operations performed on adults in Sweden. In December 2009, the registry was merged with the national health quality database, the Swedish Web-system for Enhancement and Development of Evidence-based care in Heart disease Evaluated According to Recommended Therapies (SWEDEHEART) Registry. SWEDEHEART aims to support the improvement of care and evidence-based development of therapy for coronary artery disease. The Swedish Cardiac Surgery Registry contains data on baseline characteristics, procedures, and postoperative complications (including POAF).

The objective of this study was to compare the risk of long-term (up to 9 years postdischarge) clinical outcomes in patients with and without POAF, controlling for baseline characteristics, after open-chest cardiac surgery using real-world data from SWEDEHEART and other Swedish databases with complete national coverage.

## Methods

### Study Design

**Figure 1:**
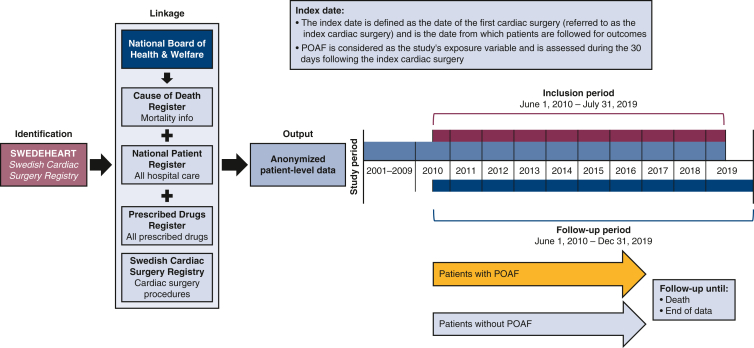
Study design. *SWEDEHEART*, Swedish Web-system for Enhancement and Development of Evidence-based care in Heart disease Evaluated According to Recommended Therapies; *POAF*, postoperative atrial fibrillation.

This observational, retrospective cohort study used pseudonymized patient data from the SWEDEHEART Swedish Cardiac Surgery Registry enriched with data from the National Patient Register, the Prescribed Drug Register, and the Cause of Death Register (Figure 1). The overall study period was from January 1, 2001, to December 31, 2019.

### Study Population

Included patients were adults aged 55 to 90 years with open-chest coronary artery bypass graft surgery (CABG) and/or valvular surgery in the SWEDEHEART Registry between June 1, 2010, and July 31, 2019. Included surgeries were isolated CABG, isolated aortic valve repair or replacement surgery, aortic valve surgery + CABG, isolated mitral valve repair or replacement surgery, mitral valve surgery + CABG, and any of the aforementioned surgeries with or without tricuspid repair/replacement (isolated tricuspid was not an inclusionary surgery). If a patient had several registered open-chest CABG or valvular surgeries, the index date was the date of the first such surgery.

Patients were excluded if they met any of the following criteria: they had a previous diagnosis of persistent or permanent AF before index cardiac surgery (patients with this history were excluded due to their likelihood of undergoing a concomitant maze or ablation procedure to treat the preexisting AF), they filled at least 1 prescription of class I or class III antiarrhythmic drug within 3 months before the index date (use of class II [beta-blocker] antiarrhythmic drugs was not excluded due to the likelihood of insufficient sample size in doing so), their index cardiac surgery was defined as an emergency surgery in the SWEDEHEART Registry, they were discharged >30 days after the index cardiac surgery (patients with abnormally long hospitalizations), it was not possible to classify the exposure status (ie, POAF) due to missing data, or the patient died during the day of the index cardiac surgery. For the time-to-event analyses, patients were also excluded if they experienced the event of interest during the day of index cardiac surgery.

Ethical approval for this study was granted on July 7, 2020, by the ethical review board in Stockholm, Sweden (registration No.: 2020-02493), and the study conduct followed legal and regulatory requirements. This study was conducted with scientific purpose, value, and rigor, following generally accepted research practices described in Guidelines for Good Pharmacoepidemiology Practices issued by the International Society for Pharmacoepidemiology and Good Practices for Outcomes Research issued by the International Society for Pharmacoeconomics and Outcomes Research. AbbVie funded this study and participated in the study design, research, analysis, data collection, interpretation of data, and reviewing and approval of the publication.

### Assessments

Patients included in the study were classified as having POAF if they had new-onset AF registered as a postsurgery complication (within 30 days postoperatively) in the SWEDEHEART Registry. New-onset POAF is not registered for patients with a preexisting diagnosis of paroxysmal AF in the SWEDEHEART Registry; patients with paroxysmal AF at index date were instead classified as having POAF if they had a registered diagnosis of AF (International Statistical Classification of Diseases and Related Health Problems, 10th Revision [ICD-10]: I480, I481, I482, I483, I484, I489) within 30 days following index cardiac surgery in the Swedish National Patient Register. Patients who did not experience POAF were classified as non-POAF patients and served as controls. Long-term outcomes of interest included risk of recurrent AF, HF, chronic kidney disease (CKD), ischemic stroke, major bleed, major adverse cardiac event (MACE) (defined as any of the following: ischemic stroke, myocardial infarction, coronary revascularization [CABG/percutaneous coronary intervention], cardiovascular death, or hospitalization due to HF or unstable angina pectoris), ventricular arrhythmia, and cardiovascular (CV) and all-cause mortality (CV mortality is defined using any of the following 2 criteria: any registered death with primary cause of death related to a CV event or condition [ICD-10: I-chapter]), or any death within 30 days of an inpatient CV event (ICD-10: I-chapter). Coding and extended definitions are presented in Table E1. Long-term outcomes were compared between patients who experienced POAF and those who did not.

### Statistical Analysis

Data extraction was conducted by the Uppsala Clinical Research Center and the Swedish National Board of Health and Welfare; data were extracted from SWEDEHEART's Swedish Cardiac Surgery Registry. Long-term CV outcomes were analyzed by time-to-first-event methods (Kaplan-Meier analysis) from index cardiac surgery to 9 years. Follow-up started from index cardiac surgery date for all outcomes except recurrent AF; follow-up for recurrent AF started 30 days after index cardiac surgery to avoid overlap with exposure of POAF. Incidence rates and 95% CI were reported by POAF exposure status, and cumulative incidence plots were generated for each CV outcome. Risk differences in CV events by POAF exposure were estimated using multivariable Cox regression, were controlled for baseline characteristics, including age, gender, and preoperative comorbidities (eg, HF, hypertension, stroke, diabetes, paroxysmal AF, and CKD), and were reported as hazard ratios (HRs) with 95% CI. Statistical analyses were performed using R software version 4.2.1 (R Foundation for Statistical Computing) and Rstudio 2022.02.4.^15^

## Results

### Patient Characteristics

**Figure E1:**
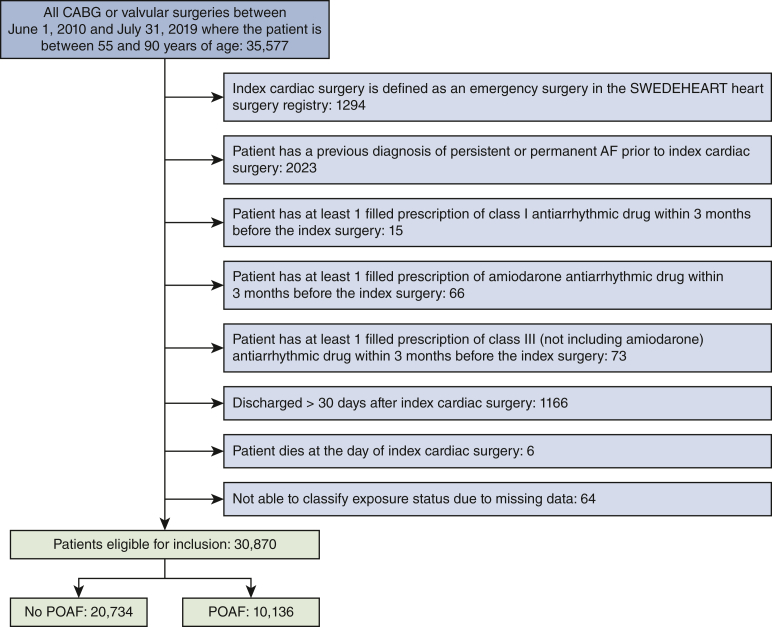
Patient attrition table. *CABG*, Coronary artery bypass graft surgery; *SWEDEHEART*, Swedish Web-system for Enhancement and Development of Evidence-based care in Heart disease Evaluated According to Recommended Therapies; *POAF*, postoperative fibrillation.

A total of 30,870 eligible patients (mean age, 69.2 years) were included in the study (Figure E1). All patients were followed up for a median of 4.6 years, and 32.8% developed POAF within 30 days postsurgery (control, n = 20,734; POAF, n = 10,136) (Table 1). Patients with POAF were slightly older, had higher proportions of valve and combination surgeries, and were more likely to have preoperative paroxysmal AF, hypertension, HF, and renal failure.

**Table 1:** Baseline characteristics

Characteristic	Control	POAF	*P* value
Patient population	20,734 (67.2)	10,136 (32.8)	–
Age (y)	69.17 ± 7.5	71.32 ± 7.1	<.01
Sex			.997
Male	15,610 (75.3)	7632 (75.3)	
Female	5124 (24.7)	2504 (24.7)	
Index surgery			<.01
CABG	13,482 (65.0)	5605 (55.3)	–
Valve	5086 (24.5)	2985 (29.4)	–
CABG + valve	2166 (10.4)	1546 (15.3)	–
Preoperative comorbidities			
Paroxysmal AF	980 (4.7)	1239 (12.2)	<.01
Diabetes	5865 (28.2)	2673 (26.4)	<.01
Hypertension	14,530 (70.1)	7452 (73.5)	<.01
Heart failure	3854 (18.6)	2103 (20.8)	<.01
Prior ischemic stroke	1177 (5.7)	611 (6.0)	.224
Renal failure	1268 (6.1)	827 (8.2)	<.01
Chronic obstructive pulmonary disease	2289 (11.0)	1173 (11.6)	.169
Baseline CHA_2_DS_2_-VASc	2.99 ± 1.5	3.23 ± 1.5	<.01
Baseline Charlson comorbidity index	1.05 ± 1.6	1.15 ± 1.6	<.01

Values are presented as n (%) or mean ± SD. *POAF*, Postoperative atrial fibrillation; *CABG*, coronary artery bypass graft surgery; *AF*, atrial fibrillation; *CHA*_*2*_*DS*_*2*_*-VASc*, congestive heart failure, hypertension, age ≥75 (doubled), diabetes, stroke (doubled), vascular disease, age 65 to 74 and sex category (female).

### Long-Term CV Outcomes

**Figure 2:**
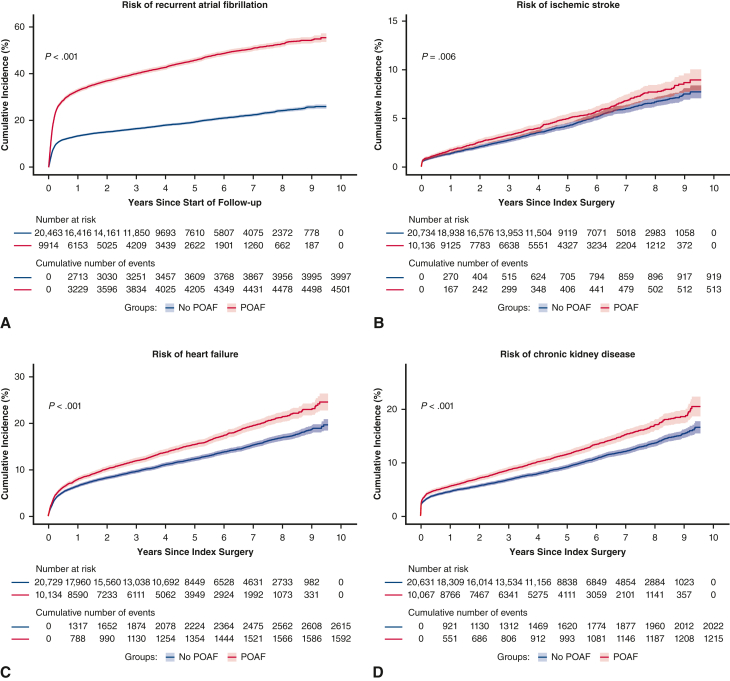

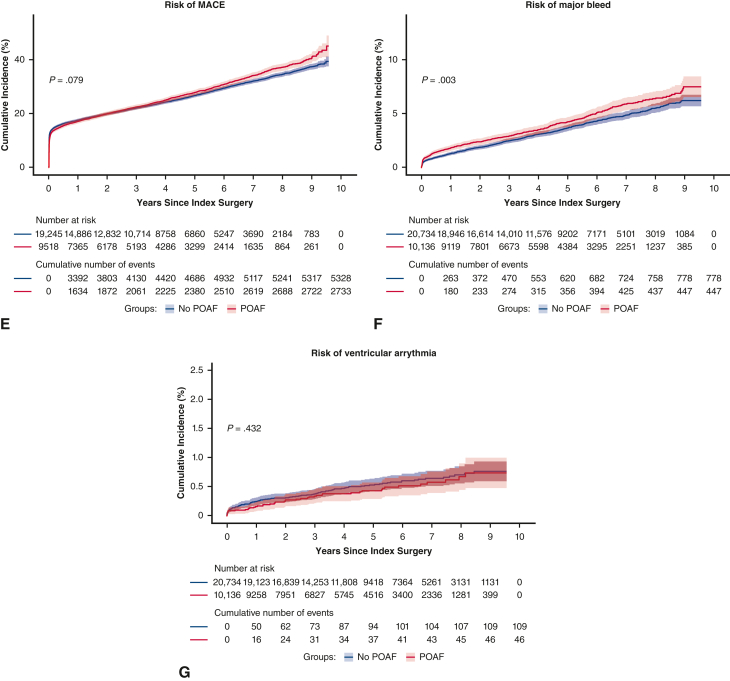
A-G, Cumulative incidence functions for cardiovascular-related outcomes. Data shown are unadjusted. *Shaded bars* represent 95% CI. *POAF*, Postoperative atrial fibrillation; *MACE*, major adverse cardiovascular event.

**Figure 3:**
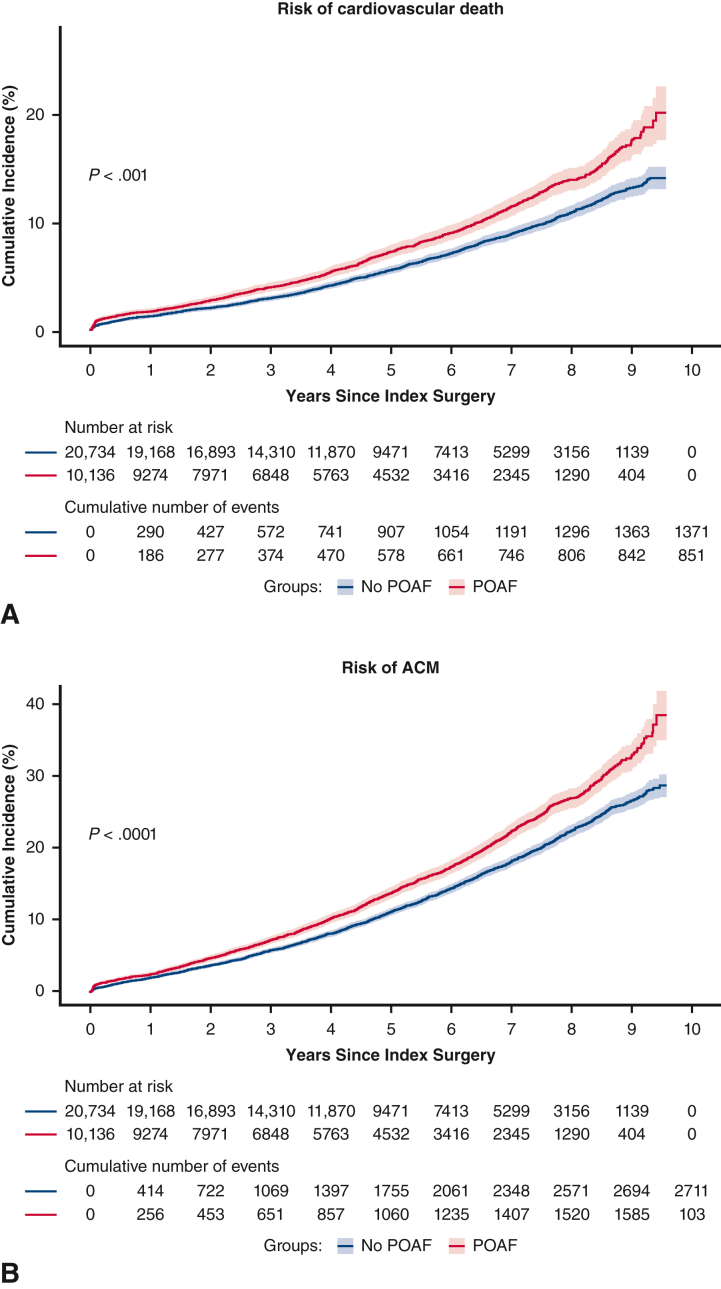
A and B, Cumulative incidence functions (with 95% CI) for cardiovascular and all-cause mortality. Data shown are unadjusted. *Shaded bars* represent 95% CI. *POAF*, Postoperative atrial fibrillation; *ACM*, all-cause mortality.

In unadjusted analyses, risk of each of the long-term outcomes, with the exception of MACE and ventricular arrhythmia, was significantly higher in the POAF cohort than control. After adjustment, patients with POAF had a significantly higher risk of the following outcomes versus patients in the control group (*P* < .01) (Table 2): recurrent AF (HR, 2.30; 95% CI, 2.21-2.41), HF (HR, 1.17; 95% CI, 1.10-1.25), CKD (HR, 1.15; 95% CI, 1.07-1.24), all-cause mortality (HR, 1.11; 95% CI, 1.04-1.18), and CV mortality (HR, 1.16; 95% CI, 1.06-1.26). POAF was associated with a numerically higher risk of ischemic stroke and major bleed, but these findings were not statistically significant after adjustment. Statistically significant differences for patients with POAF versus patients in the control group were observed for the cumulative incidence risk of AF (*P* < .0001), ischemic stroke (*P* = .006), major bleed (*P* = .003), HF (*P* < .0001), CKD (*P* < .0001), and all-cause mortality (*P* < .0001), but not MACE or ventricular arrhythmia (Figures 2 and 3). Incidence rates of long-term CV outcomes are summarized in Table E2.

**Table 2:** Risk of long-term cardiovascular outcomes associated with postoperative atrial fibrillation

CV-related outcomes	Unadjusted hazard ratio (95% CI)	Adjusted hazard ratio (95% CI)
Recurrent AF	2.79 (2.68-2.91)∗	2.30 (2.21-2.41)∗
Ischemic stroke	1.18 (1.06-1.32)∗	1.07 (0.96-1.20)
Heart failure	1.29 (1.21-1.38)∗	1.17 (1.10-1.25)∗
Chronic kidney disease	1.28 (1.19-1.37)∗	1.15 (1.07-1.24)∗
MACE	1.04 (1.00-1.09)	0.99 (0.94-1.03)
Major bleed	1.21 (1.08-1.36)∗	1.11 (0.98-1.25)
Ventricular arrhythmia	0.88 (0.63-1.25)	0.90 (0.64-1.28)
All-cause death	1.27 (1.19-1.35)∗	1.11 (1.04-1.18)∗
CV death	1.33 (1.22-1.45)∗	1.16 (1.06-1.26)∗

*CV*, Cardiovascular; *CI*, confidence interval; *AF*, atrial fibrillation; *MACE*, major adverse cardiovascular event.

∗ Statistically significant at *P* < .01.

## Discussion

**Figure 4:**
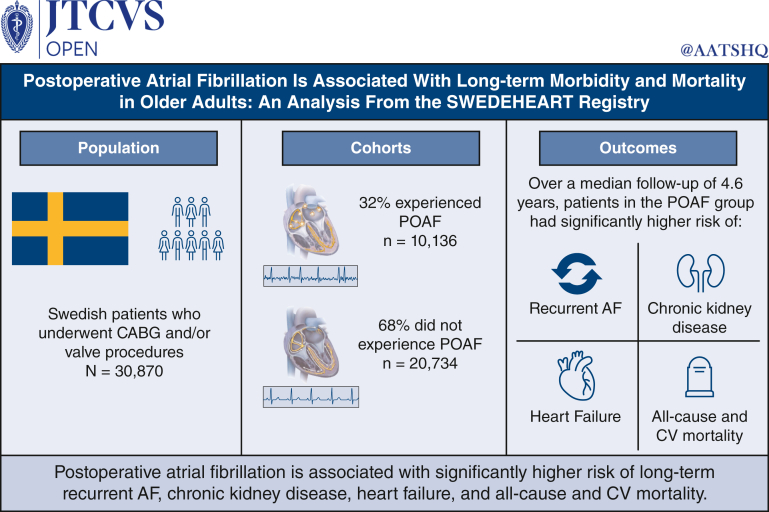
Study design, key findings, and implications of a retrospective cohort study using data from the Swedish Web-system for Enhancement and Development of Evidence-based care in Heart disease Evaluated According to Recommended Therapies (SWEDEHEART) Swedish Cardiac Surgery Registry and National Board of Health and Welfare to compare risk of long-term clinical outcomes in patients with and without postoperative atrial fibrillation (*POAF*) following open-chest surgery. *CABG*, Coronary artery bypass graft; *AF*, atrial fibrillation; *CV*, cardiovascular.

In this analysis of comprehensive, real-world data from the Swedish Cardiac Surgery Registry, POAF in older adults was associated with a significantly higher risk of long-term recurrent AF, HF, CKD, and all-cause and CV mortality after adjustment for baseline characteristics (Figure 4). POAF was also associated with a numerically higher risk of ischemic stroke and major bleed, but these findings were not statistically significant after adjustment; the longer follow-up time and selected adjustment variables may play a role in this finding. Whereas limited information is available regarding the association of POAF with longer-term clinical outcomes,^8^ the results of this study lend support to existing literature. For example, in another study based on data from the SWEDEHEART Registry, POAF after isolated CABG was associated with significantly increased risk of ischemic stroke (HR, 1.18; 95% CI, 1.05-1.32).^16^ In a single-center database study, POAF predicted future AF, HF hospitalization, and overall mortality, but not transient ischemic attack or ischemic stroke.^17^ A study of patients without a history of AF undergoing CABG showed that POAF was the strongest predictor of late AF (adjusted risk ratio, 8.31; 95% CI, 4.20-16.43).^7^

Historically, findings linking POAF and stroke have been inconsistent.^18^ Confounding factors may include the use of anticoagulant treatment, which could reduce the risk of stroke.^18^ Anticoagulant treatment has become more engrained in clinical practice for stroke prophylaxis, especially as new factor Xa inhibitors have been introduced.^19^ This shift in treatment pattern may be partially responsible for why a significant difference in the risk for stroke among patients with and without POAF was not observed in our study.

We recognize that this study shares many similarities with the study by Taha and colleagues,^16^ which utilized the same Swedish data sources. However, we emphasize several key differences, the largest being the different types of included procedures. Taha and colleagues^16^ analyzed isolated CABG cases, whereas the current study employed a more general cardiac surgery viewpoint. The report from Taha and colleagues^16^ found that early initiation of oral anticoagulant treatment in patients with POAF was associated with increased risk for bleeding, but not with reduced risk for stroke. In our study, ischemic stroke and major bleed were the 2 lowest-frequency outcomes (aside from ventricular arrhythmia). Although there is a numerically higher risk of major bleed and ischemic stroke associated with POAF, sample size and the low number of events likely prevented the influence of these factors from being statistically significant as individual outcomes. As anticoagulant use becomes more commonplace in medical practice, it may be more difficult to observe significant differences in stroke risk. We highlight these differences to note the importance of reexploring findings from previous studies on similar data sources using similar but not identical methods. Whereas most findings are consistent, we did uncover some small but important differences that simultaneously add to the total understanding of the late negative effects associated with POAF and uncover new directions for future research.

Strengths of this study include the wide range of long-term outcomes evaluated and the long follow-up time (up to 9 years). The comprehensive nature of the Swedish databases adds to the robustness of the study. Limitations include the retrospective, observational study design, which is subject to inherent biases in data collection and unknown confounders. In addition, there was limited ability to control for certain characteristics that are not well captured in retrospective data, such as laboratory or procedural findings as well as AF burden or severity (outside of what is documented by a diagnosis code). We acknowledge that mixing CABG and valve patients may have introduced confounding effects due to potential differences in long-term oral anticoagulant use between the 2 groups, reliable long-term data for which were not available. Exclusion of concomitant surgical ablation and/or left atrial appendage closure procedures may not have been reliable during the early years of the study because cardiac surgery standards and SWEDEHEART Registry data variables have evolved over time. There is some risk of underestimating disease burden due to incomplete data collection; however, the data are known to have a high degree of completeness. Immortal time bias may be possible due to the determination of the exposure of POAF being made up to 30 days postoperatively (ie, after index). This could lead to an overestimation of the effect and should be taken into account. However, all patients except those with paroxysmal AF at index surgery had exposure classified between surgery and discharge, which on average was 6.64 days; thus, the potential bias should be small. Finally, data were collected in a single country, which may limit generalizability to a global population, although some findings may be generalizable to other countries where patient characteristics and treatment practices are similar.

## Conclusions

In this analysis of real-world data from comprehensive Swedish databases, over a median of 4.6 years of follow-up after cardiac surgery and after adjustment for baseline risk factors, POAF was associated with a significantly higher risk of long-term recurrent AF, HF, CKD, and all-cause and CV mortality, and a numerically higher risk of major bleed and ischemic stroke.

## Conflict of Interest Statement

Mr Lilja and Mr Banefelt are employees of Quantify Research, a contract research organization that provides consultancy services to the pharmaceutical industry. Mr Park and Drs Shah, Leaback, and Ferguson are employees of AbbVie and may hold AbbVie stock. Dr Friberg is chairman of the Swedish Cardiac Surgery Registry and reports having received financial compensation for consultancy services to AbbVie regarding interpretation of Swedish Web-system for Enhancement and Development of Evidence-based care in Heart disease Evaluated According to Recommended Therapies (SWEDEHEART) data.

The *Journal* policy requires editors and reviewers to disclose conflicts of interest and to decline handling manuscripts for which they may have a conflict of interest. The editors and reviewers of this article have no conflict of interest.

## Figures and Tables

**Table E1:** Outcome definitions and International Statistical Classification of Diseases and Related Health Problems, 10th Revision (ICD-10) codes

Outcome	Definition	Measurement period	ICD-10 codes	NCSP/nonsurgical procedure codes, any position	Primary/secondary diagnosis or cause of death	Inpatient/outpatient visit recorded in the NPR	Registry
Recurrent atrial fibrillation	Primary and secondary diagnosis associated with health care encounters, including hospitalizations and specialist outpatient visits, or registered DC cardioversion after 6 mo from index cardiac surgery	From start of f/u to registered event or censoring event (end of data, death)	I480, I481, I482, I483, I484, I489	After 6 mo from index cardiac surgery: DF010, DF026, DF027	Primary AND Secondary	Inpatient AND Outpatient	NPR
Ischemic stroke	Primary diagnosis associated with hospital admission	From start of f/u to registered event or censoring event (end of data, death)	I61, I63, I64, I679, G459	No	Primary	Inpatient	NPR
Heart failure	Primary with health care encounters, including hospitalizations and specialist outpatient visit	From start of f/u to registered event or censoring event (end of data, death)	I50	No	Primary	Inpatient AND Outpatient	NPR
Chronic kidney disease	Primary and secondary diagnosis associated with health care encounters, including hospitalizations and specialist outpatient visit	From start of f/u to registered event or censoring event (end of data, death)	N17, N18, N19, I120, I131, I132, Z49, Z992	No	Primary AND Secondary	Inpatient AND Outpatient	NPR
All-cause death	Any registered death	From start of f/u to registered event or censoring event (end of data)	No	No	Any death registered	NA	CDR
CV-related death	Any registered death with primary cause of death related to a cardiovascular event or condition	From start of f/u to registered event or censoring event (end of data, death)	Primary cause of death: I00-I99	No	Primary AND Secondary/ Any death registered	Inpatient (for any death within 30 d of an inpatient CV event)	NPR, CDR
MACE, composite outcome, including • Ischemic stroke • Myocardial infarction • Coronary revascularization • Heart failure • Hospitalization due to unstable angina pectoris • CV-related death	Primary diagnosis/procedure associated with hospital admission	From start of f/u to registered event or censoring event (end of data, death)	• Ischemic stroke: Same as above • Myocardial infarction: I21, I22 • Heart failure: I50 only associated with hospitalization • Hospitalization due to unstable angina pectoris: I200 • CV-related death: Same as above	• Coronary revascularization: FNA0, FNA00, FNA10, FNA20, FNA96, FNB00, FNB20, FNB96, FNC10, FNC20, FNC30, FNC40, FNC50, FNC60, FNC96, FND10, FND20, FND96, FNE00, FNE10, FNE20, FNE96, FNF00, FNF10, FNF20, FNF30, FNF96; PCI: FNG0, FNG00, FNG02, FNG05, FNG06, FNG10, FNG30, FNG96	Primary/any death registered	Inpatient	NPR, CDR
Major bleed	Primary diagnosis associated with hospital admission	From start of f/u to registered event or censoring event (end of data, death)	I60, I61, I62, K250, K252, K254, K260, K262, K264, K270, K272, K274, K280, K282, K290, K920, K921, K922, D62, J942, H113, H356, H431, N02, R04, R31, R58	No	Primary	Inpatient	NPR
Ventricular arrhythmia	Primary diagnosis associated with hospital admission	From start of f/u to registered event or censoring event (end of data, death)	I472	No	Primary	Inpatient	NPR

*ICD-10*, International Statistical Classification of Diseases and Related Health Problems, 10th Revision codes; *NCSP*, The Nomesco Classification of Surgical Procedures; *NPR*, National Patient Registry; *DC*, direct current; *f/u*, follow-up; *CDR*, Cause of Death Registry; *CV*, cardiovascular; *MACE*, major adverse cardiac event.

**Table E2:** Incidence rates of long-term cardiovascular outcomes

Outcome	Incidence rate∗ (95% CI)	
	No POAF	POAF
Recurrent AF	4.87 (4.72-5.03)	15.33 (14.89-15.79)
Ischemic stroke	0.95 (0.89-1.02)	1.13 (1.03-1.23)
Heart failure	2.89 (2.78-3.00)	3.77 (3.59-3.96)
Chronic kidney disease	2.16 (2.07-2.26)	2.79 (2.64-2.96)
MACE	7.18 (6.98-7.37)	7.66 (7.37-7.95)
Major bleed	0.80 (0.75-0.86)	0.98 (0.89-1.07)
Ventricular arrhythmia	0.11 (0.09-0.13)	0.10 (0.07-0.13)
All-cause death	2.74 (2.64-2.84)	3.42 (3.25-3.59)
CV death	1.38 (1.31-1.46)	1.81 (1.70-1.94)

*CI*, Confidence interval; *POAF*, postoperative atrial fibrillation; *AF*, atrial fibrillation; *MACE*, major adverse cardiovascular events; *CV*, cardiovascular.

∗ Incidence rate is events per 100 person-years.
